# The impact of transgenesis on mosquito fitness: A review

**DOI:** 10.3389/finsc.2022.957570

**Published:** 2022-09-30

**Authors:** Padukka Vidanelage Desha Dilani, Ranil Samantha Dassanayake, Brij Kishore Tyagi, Yasanthi Illika Nilmini Silva Gunawardene

**Affiliations:** ^1^ Department of Chemistry, Faculty of Science, University of Colombo, Colombo, Sri Lanka; ^2^ Sponsored Research & Industrial Centre, VIT University, Vellore (TN), India; ^3^ Molecular Medicine Unit, Faculty of Medicine, University of Kelaniya, Ragama, Sri Lanka

**Keywords:** mosquito, fitness, transgenic, transgene, disease

## Abstract

Transgenic mosquitoes developed by genetic manipulation, offer a promising strategy for the sustainable and effective control of mosquito-borne diseases. This strategy relies on the mass release of transgenic mosquitoes into the wild, where their transgene is expected to persist in the natural environment, either permanently or transiently, within the mosquito population. In such circumstances, the fitness of transgenic mosquitoes is an important factor in determining their survival in the wild. The impact of transgene expression, insertional mutagenesis, inbreeding depression related to laboratory adaptation, and the hitchhiking effect involved in developing homozygous mosquito lines can all have an effect on the fitness of transgenic mosquitoes. Therefore, real-time estimation of transgene-associated fitness cost is imperative for modeling and planning transgenic mosquito release programs. This can be achieved by directly comparing fitness parameters in individuals homozygous or hemizygous for the transgene and their wild-type counterparts, or by cage invasion experiments to monitor the frequency of the transgenic allele over multiple generations. Recent advancements such as site-specific integration systems and gene drives, provide platforms to address fitness issues in transgenic mosquitoes. More research on the fitness of transgenic individuals is required to develop transgenic mosquitoes with a low fitness cost.

## Introduction

Mosquito-borne diseases, including dengue, malaria, yellow fever, and chikungunya, continue to have substantial health, social, and economic burdens on the human population worldwide. Each year nearly 700 million people are affected by mosquito-borne diseases, and more than 1 million die from mosquito-borne diseases (1). In the absence of effective vaccines, reliable therapeutics or solid diagnostics, and proper clinical management strategies, disease control primarily relies on mosquito vector control (2, 3). However, in the past few decades, traditional mosquito control methods such as the removal of mosquito breeding sites, the application of insecticides and mosquito repellents, and the introduction of biological agents that act as natural enemies have proven largely ineffective (2, 4–6). With the advent of modern biotechnology, genetic manipulation of vector mosquitoes has paved the way for multiple novel approaches that can lead to the development of alternative strategies to alleviate the burden of mosquito-borne diseases. Numerous genetic tools required for the genetic manipulation of mosquitoes have been developed over the last few decades, with the result that exogenous genes can now efficiently be transformed into the germline of mosquitoes through microinjection (7). Furthermore, the discovery of phenotypic markers and the characterization of tissue-specific and stage-specific promoter sequences, as well as the identification of novel effector genes, play a significant role in enriching the genetic toolbox available for transgenic mosquito studies (8–10).

In general, genetic control of the mosquito can be achieved through either population replacement (self-sustaining) or population reduction (self-limiting) approaches (11). Population reduction strategies involve the release of genetically sterile mosquitoes carrying an effector gene that impairs offspring production in a targeted mosquito population. The release of insects carrying a dominant lethal gene (RIDL) is the most widely used strategy based on this approach. Recent advancements in RIDL have resulted in the development of several transgenic mosquitoes, some of which are currently being tested in selected field studies and others which are available to be used in the wild (11–13). Population replacement, on the other hand, uses transgenic mosquitoes that are refractory to a given pathogen to replace the wild population (14). This employs RNA interference (RNAi) mechanisms, artificial peptides, and many other anti-pathogen effector genes (15–17) that have shown promising results in combating mosquito-borne diseases. Furthermore, these replacement strategies can also be combined with gene drives to speed transgene spread in the population. All these control strategies, in turn, involve the release of transgenic mosquitoes into the wild to introduce the transgene into the mosquito population either permanently or transiently (18). The success of the strategies, however, is entirely dependent on the performance of the released mosquitoes, especially in terms of mating and reproductive performance. Therefore, before attempting to implement release programs, it is essential to evaluate whether genetic modification itself and/or mass rearing in the laboratory may impose a fitness cost. Otherwise, the performance of the transgenic mosquitoes will be substantially reduced and the spread of the transgene into the wild may be extremely difficult (14). Therefore, in this review, we discuss the possible sources of fitness cost/fitness load associated with transgenic mosquitoes, and the recent advancements in estimating transgenic mosquito fitness costs using various methods. This will be helpful in developing transgenic mosquitoes to combat mosquito-borne viral diseases.

## Sources of fitness cost in transgenic mosquitoes

Fitness in transgenic mosquitoes is defined as relative success in terms of survival and reproduction resulting in the transmission of the transgenes to subsequent generations (19). There are two categories of fitness: (i) developmental fitness and (ii) reproductive fitness. Developmental fitness is a measure of ability to adapt and survive in the natural environment, whereas reproductive fitness measures the ability to pass genes on to the next generations (20, 21). Since transgenic individuals are evolutionary novelties, they are frequently less fit than wild-type counterparts due to the following reasons (i) the impact of transgene products: (i) transgene products; (ii) the position effect and insertional mutagenesis; (iii) inbreeding depression resulting from laboratory adaptation and the fixation of deleterious recessive alleles during the process of creating transgenic lineage; and (iv) the hitchhiking effect resulting from transgene insertion to a point near a deleterious recessive gene in homozygous individuals (18).

### Impact of transgene product

Genetic mosquito control approaches involve the utilization of multiple transgenes, with the aim of providing effective means of mosquito control. The most common transgenes include effector genes, fluorescence marker genes, and transposase genes to achieve transposon-mediated transformation. In addition, the RIDL strategy, in particular, uses a repressible transactivator gene to activate conditional lethality (22). Expression of these exogenous genes may be detrimental or have an adverse impact on the fitness of the transgenic individual, for example if the gene product is toxic or gene translation usurps the resources needed for normal reproductive functions, or if transgene expression imposes a heavy genetic load on mosquitoes (10). Hence, it is highly preferable to select effective promoters and gene sequences that minimize the fitness cost. In this respect, to improve the fitness of transgenic insects, the artificial promoter *3xP3* has been designed to drive the fluorescence marker gene expression in the ommatidium of insects’ eyes (23) and then restrict the expression of fluorescence protein in the eye tissue to minimize the impact on fitness. In contrast to promoters that drive tissue-specific expression, ubiquitous promoters that drive the expression in all mitotically active tissues throughout development can have an adverse impact on fitness. The *5C* promoters of *Drosophila melanogaster* and *Hr5E1* (baculovirus *IE1* promoter and *Hr5* enhancer) are the most common ubiquitous promoters used in mosquito studies (24).

Moreover, transgene expression in transgenic mosquitoes can be either intentional or off-target. Particularly in female-specific RIDL (fsRIDL) mosquitoes, transgene expression is intended to be lethal only to females reared in the absence of antidote tetracycline (“off-tet”), whereas off-target expression could have a deleterious impact on male mosquitoes. Similarly, females may also experience deleterious effects if transgene expression is not suppressed below a harmful level, even when tetracycline is present. Female-specific and stage-specific conditional expression in these systems is achieved by selecting inducible promoters with low leaky basal expression and effector/lethal genes. These gene products act in a stoichiometric manner rather than in a catalytic manner (18, 19). For example, a system based on the flightless *Aedes aegypti* fsRIDL strain has been developed to control mosquitoes. In this system, the lethal gene is driven under the control of a female-specific indirect flight muscle promoter from the *Ae. aegypti Actin 4* gene (22) and selectively kills the targeted subpopulation while minimizing harm to off-target subpopulations. In addition, female-specific and stage-specific promoters are now widely used in population replacement strategies as well as to drive the expression of different anti-pathogen effector genes. A bloodmeal-inducible promoter sequence from *Ae. aegypti* carboxypeptidase A gene (*AeCPA*) is the most common example, and is used to drive the expression of the anti-dengue viral effector gene to inhibit viral replication (15, 25).

Rapid technological advancements in DNA sequencing, protein characterization, and bioinformatics provide unprecedented opportunities to construct sex-specific and tissue-specific transcriptomic profiles of different mosquitoes. Transcriptomic atlases have been created for several species of *Anopheles* (26–28) and *Ae. aegypti* (29), and these facilitate the identification of genes with highly tissue/cell-specific expression patterns. Such genes are ideal candidates for use in mosquito control, and the promoters of such genes can be employed to selectively drive transgene expression and thus minimize any potential fitness costs.

### Position effect and insertional mutagenesis

The use of transposable elements for germline transformation generally gives rise to position effect and insertional mutagenesis, owing to the random pattern of genome integration. The genomic position of transgene insertion can have a significant impact on the fitness of transgenic mosquitoes. The level of transgene expression can be influenced by the genomic sequences of enhancers and silencers in the vicinity of the transgene, which can even lead to transgene expression in different tissues and/or stages than those intended (i.e., off-target expression) (30). This effect is specifically problematic in highly regulated expression systems such as RIDL. Its expression is based on a tetracycline-repressible system, which requires a high level of expression in the absence of tetracycline and a low level of expression in the presence of tetracycline. However, the heterochromatic region at which the insertion occurs can lead to a low level of transgene expression (31) and, as a result, the undesired gene expression caused by the positional effects can lead to a reduction in fitness in transgenic individuals. Consequently, the fitness cost may vary between different transgenic lines carrying the same transgene depending on the position of the insertion (18). These position effects can be minimized by flanking the transgenes with insulators or DNA boundary elements, as they can block the unwanted effect of nearby enhancers and silencers and prevent the effect of heterochromatin (32).

In addition to the positional effect, the integration of transgenes into an open reading frame or regulatory sequences of an essential endogenous gene (insertional mutagenesis) can lead to partial or complete disruption of gene function, resulting in either reduced fitness or recessive lethality (33). However, insertional mutagenesis is found to be recessive for many genes, presumably because the genes are integrated either into the non-coding region or into a region of genes that are not essential for survival (34). In this respect, transgenic lines created by disrupting a coding sequence (IV, homolog of *D. melanogaster* chaoptin precursor) and without disrupting the coding sequence (V_D12_) of *Anopheles stephensi* have shown similar performance in transgene persistence (35). To mitigate these undesirable outcomes, new, efficient tools have been developed. Site-specific transgene integration is one such system that prevents insertional mutagenesis due to random integration using an alternative system to transposable elements. Among them, the phage phiC31 system and a modified CRE–*lox* mechanism is widely used in mosquitoes to preclude insertional mutagenesis on transgene expression (36). Furthermore, the use of insulators derived from the *Drosophila* gypsy transposon, together with a site-specific phiC31 system, has shown more stabilized and precise transgene expression in the malaria vector mosquito, *An. stephensi (* 32 *).* Therefore, the location of the transgene is a major factor of concern, and the fitness cost associated with transgene expression could be either mitigated or compounded based on the strategies used to protect the transgene from the effects of its genomic environment.

### Laboratory adaptation and inbreeding depression

Another way in which fitness can be affected is through inbreeding depression related to laboratory adaptation. In general, transgenic approaches involve rearing and releasing mosquitoes into the wild. Therefore, large numbers of genetically modified mosquitoes need to be reared in laboratories before release. However, laboratory rearing itself may impose on mosquitoes a fitness load that is not experienced by mosquitoes in their natural habitats. For example, in the wild, mosquitoes, rely on olfactory cues not only to find food or blood sources but also to find mates and breeding sites (37, 38). The responses of mosquitoes to odorants directly affect their reproductive success and their life history, which in turn determines their evolutionary fitness (39). Mosquitoes reared in a laboratory are maintained in inherently artificial conditions, and these colonized mosquitoes experience a different set of selective pressures than the wild mosquitoes (40). In laboratories, they are maintained at a controlled temperature, humidity, and photoperiod, provided with abundant nutrients, and reared in discrete generations (41). This may result in a loss of sensitivity to such olfactory cues, leading to a significant fitness cost for the individuals. Furthermore, laboratory-colonized mosquitoes are maintained at high densities owing to space limitation, which ultimately leads to intense male–male competition and alters courtship behavior (42–44). In addition, the lack of selective pressures in these environments will lead to a reduced ability to survive at extreme temperatures or in dry conditions, or to survive periods of starvation (45) or loss of resistance to insecticide (46, 47). Therefore, laboratory-colonized mosquitoes often have low adaptive potential compared with their natural counterparts, which often results in a reduction in genetic variation followed by inbreeding depression (48).

In addition to laboratory adaptation, inbreeding depression can also occur while developing transgenic lines. Transgenic lines typically originate from a single transformed mosquito crossed with at least one or a few wild-type mosquitoes (35). Any deleterious recessive mutation associated with the initial insertion has a high chance of being fixed (33). However, this inbreeding depression can be diminished through successive outcrossing of transgenic mosquitoes to the more genetically diverse wild-type strain.

### Hitchhiking effect

**Figure 1:**
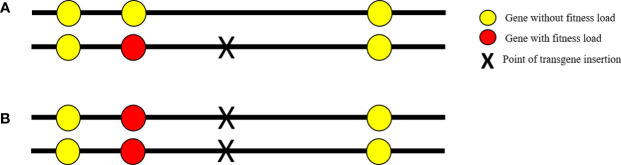
The hitchhiking effect. **(A)** Chromosomal loci hemizygous for a transgene insert (black X); **(B)** chromosomal loci homozygous for the transgene insert. Red circles represent the deleterious recessive gene. During transgenesis, the transgene may integrate into the vicinity of a deleterious recessive allele and, when the transgene is made homozygous, any nearby recessive gene will also become homozygous in a process known as the “hitchhiking effect.”.

The hitchhiking effect can also negatively affect the fitness of transgenic mosquitoes when they are made homozygous. Many organisms often carry deleterious recessive alleles that express all their harmful effects only when they are homozygous. During mosquito transgenesis, if the transgene integrates into the vicinity of a deleterious recessive allele, the subsequent inbreeding may generate homozygous individuals and any nearby recessive allele may also become homozygous, conferring reduced fitness, which is known as the hitchhiking effect (**Figure 1**) (19). This effect can be alleviated through the repeated crossing of transgenic individuals with their wild-type counterparts and selecting the best hemizygous lines before establishing the homozygous transgenic line. Repeated backcrossing allows the recombination of the deleterious recessive genes and causes the dissociation of the recessive allele from the transgene, thereby increasing transgene stability (49).

## Assessment of the fitness on transgenic mosquitoes

In general, wild-type mosquitoes possess an evolutionary advantage in terms of fitness over genetically modified mosquitoes. Fitness cost often makes transgenic mosquitoes less fit to survive in the natural environment, suggesting that the genetically modified individuals must experience no fitness cost or only a very low fitness cost if they are to survive and compete with their wild-type counterparts. Therefore, it is imperative to evaluate the fitness of transgenic individuals to enable the feasibility of using transgenic individuals in mosquito control programs to be determined. A range of fitness studies have evaluated the fitness of transgenic mosquitoes, particularly in *Ae. aegypti* and *An. stephensi* (**Table 1**). The majority of fitness studies have found significant fitness load in some transgenic lines as a result of one or more of the aforementioned sources of a fitness cost. The assessment of fitness can be done in multiple ways.

**Table 1:** Fitness studies on transgenic mosquitoes.

Number	Species	Type of study	Name of the transgenic line	Transposon system	Promoter	Gene product	Zygosity of the transgenic line	Fitness cost	Reference(s)
**1**	*Ae. aegypti*	A	EGFP	*Hermes*	*Actin 5C*	EGFP	Homozygous	Yes	(24)
		A	auto*Hermes*	*Hermes*	*Hsp70* *Actin 5C*	*Hermes* transposase EGFP	Homozygous	Yes	(24)
		A	pBacMOS	*piggyBac*	*Hsp70* *3xP3*	*MOS1* transposase EGFP	Homozygous	Yes	(24)
**2**	*Ae. aegypti*	A	OX513A	*piggyBac*	*Hsp70/tetO* *Actin 5C*	tTAV DsRed	Homozygous	Yes	(50)
	*An. gambiae*	A	FREP1-KOs	*phiC31*-mediated site-specific integration system	*U6 snRNA pol III* *Vasa 2*	gRNA (FREP1 knockout) Cas9	Homozygous	Yes	(51)
**3**	*An. stephensi*	B and C	PLA_2_	*piggyBac*	*AgCP* *3xP3*	PLA_2_ EGFP	Heterozygous	Yes	(52)
				*piggyBac*	*AgCP* *3xP3*	SM1 EGFP	Heterozygous	No	(52)
**4**	*Ae. aegypti*	B	SL1161	*piggyBac*	*AeCPA*	RNAi	Heterozygous	Yes	(21, 25)
**5**	*An. stephensi*	C	IV	*Minos*	*Actin 5C*	EGFP	Homozygous	Yes	(35)
			VD12	*Minos*	*Actin 5C*	EGFP	Homozygous	Yes	(35)
			ASML12	*Minos*	*Actin 5C*	EGFP	Homozygous	Yes	(35)
			MinRED1	*Minos*	*Actin 5C*	DsRed	Homozygous	Yes	(35)
**6**	*An. gambiae*	A and C	EE	*piggyBac phiC31*-mediated site-specific integration system	*3xP3*	ECFP	Homozygous	Yes	(33)
			Evida3	*piggyBac phiC31*-mediated site-specific integration system	*3xP3* *AgCPA*	DsRed AMP	Homozygous	Yes	(33)
**7**	*An. stephensi*	A and C	VD9 (double insertion)	*piggyBac*	*3xP3* *AeVg*	EGFP SM1	Homozygous	No	(53)
			VD26 (single insertion)	*piggyBac*	*3xP3* *AeVg*	EGFP SM1	Homozygous	Yes	(53)
			VD35 (single insertion)	*piggyBac*	*3xP3* *AeVg*	EGFP SM1	Homozygous	Yes	(53)

Type of study; **(A)** direct comparison of fitness parameters on homozygotes, **(B)** direct comparison of fitness parameters on hemizygotes/heterozygotes, and **(C)** evaluation of fitness in terms of changes in transgene allele frequency through cage invasion experiments.

AeVg, *Ae. aegypti* vitellogenin; AgCP, *An. gambiae* carboxypeptidase; AgCPA, *An. gambiae* carboxypeptidase A; Cas9 CRISPR-associated protein 9; CRISPR, clustered regularly interspaced short palindromic repeats; DsRed, red fluorescence protein; EGFP, enhanced green fluorescent protein gene; FREP1, fibrinogen-related protein 1; gRNA, guide RNA; Hsp70, heat shock protein 70; PLA_2_, phospholipase A_2_; tetO, tetracycline operator; tTAV, tetracycline-controlled transactivator; U6 snRNA pol III, U6 small nuclear RNA polymerase III.

### Comparison of fitness parameters on homozygous individuals

One of the most common approaches to evaluate fitness is a direct comparison of fitness parameters of individuals homozygous for the transgene to their non-transgenic counterparts. The use of homozygous lines rather than hemizygous transgenic lines has certain advantages:

1. Gene expression of effector molecules is stronger in homozygous transgenic lines than in hemizygous mosquitoes.
2. Mass rearing of mosquitoes is required for field release programs, and the use of homozygotes is easier and more efficient than with hemizygotes.
3. Quick and efficient introgression of the transgene into the population can be achieved only with homozygous individuals.
4. Fixation of recessive deleterious genes can be observed only in homozygous mosquitoes. The use of homozygous mosquitoes, therefore, enables the selection of the best transgenic lines with integration events that do not reside near the recessive deleterious genes (53).

For these reasons, it is desirable to assess fitness parameters on homozygotes and to select the fittest homozygous line from among several transgenic lines, disregarding unfit lines that are unworthy of further improvement (33). Several studies have assessed the fitness parameters of homozygotes; for example, Irvin *et al. (* 24) examined the reproductive and developmental fitness of three homozygous transgenic lines of *Ae. aegypti*, one carrying the enhanced green fluorescent protein gene (*EGFP*), one carrying a transposase gene from the *Hermes* transposable element, and one carrying a transposase gene from the *Mos1* transposable elements. This revealed a higher fitness cost in transgenic lines than in non-transgenic mosquitoes. The authors observed significantly reduced survival at all life stages across all gonotrophic cycles, whereas higher mortality was observed during the transition from eggs to the larval stage. Furthermore, reduced fecundity was observed in all transgenic lines relative to the non-transgenic strain, with the most impaired fecundity seen in *EGFP*-carrying strains. Moreover, adult longevity was lowest for two lines. Even though proven gene driving mechanisms and effector genes were not incorporated into all three transgenic lines studied, the severe reduction in fitness in Irvin *et al.*’s preliminary study suggests that being transgenic is associated with a serious fitness cost. Similarly, in another study, life history parameters, including larval mortality and development rate, adult size, and longevity, were compared in a genetically modified *Ae. aegypti* strain (OX513A) carrying a late-acting RIDL positive feedback system and unmodified, genetically similar, counterparts (50). The authors found reduced performance, in terms of larval survival and adult longevity, and reduced body size in the OX513 homozygous line compared with the unmodified counterpart. Later, Massonnet-Bruneel *et al. (* 54) compared mating competitiveness, insemination rate, and adult male longevity in homozygous *Ae. aegypti* RIDL males (OX513) and their wild-type counterparts in laboratory conditions. Despite comparable mating competitiveness, the authors observed slightly lower median longevity in newly emerged RIDL males under off-tet conditions than in the wild-type counterparts.

With the advancements in mosquito transgenesis, more recent studies of interest have employed clustered regularly interspaced short palindromic repeats (CRISPR)/CRISPR-associated protein 9 (Cas9) gene-editing tools to generate transgenic mosquitoes. In one of these investigations, Dong *et al. (* 51) used CRISPR/Cas9 to knockout the fibrinogen-related protein 1 gene (*FREP1*) in *An. gambiae*, and found that the knockout mosquitoes’ (“FREP1KO”) susceptibility to infection with the malaria parasite was profoundly suppressed. However, an assessment of the fitness of the homozygous transgenic line revealed a significant fitness cost in transgenic individuals compared with wild-type strains. They observed lower levels of blood-feeding propensity and fecundity, lower egg-hatching rates, a retarded pupation time, and reduced longevity after a blood meal (51). In a similar study, genetic knockout of actin (*AeAct-4*) and myosin (*myo-fem*) genes in *Ae. aegypti* resulted in 100% female flightless (55). Homozygous male mosquitoes, although able to fly, mate, and produce offspring, showed reduced performance compared with wild-type males (55). One common feature of the aforementioned studies is that the transgenic mosquito lines were maintained as homozygotes. The lower fitness observed in these individuals, therefore, could be due either to fitness cost associated with direct transgenesis (negative effect of transgene product and insertional mutagenesis) or to issues linked to the genetic background of the mosquito (e.g., inbreeding depression and hitchhiking of deleterious recessive genes). However, the direct comparison of fitness parameters of homozygous individuals does not distinguish between fitness cost derived directly from transgenesis and fitness cost linked to genetic background.

### Comparison of fitness parameters on hemizygous individuals

The second approach, comparing the fitness parameters of individuals hemizygous for the transgene with that of wild-type strains, resolves the limitation observed in the first approach, which relies on homozygous individuals. The use of hemizygous lines can eliminate the fitness cost derived from inbreeding depression and hitchhiking of deleterious recessive genes. This approach was followed in a recent laboratory trial (25) in *Ae. aegypti* in which the fitness of hemizygous transgenic individuals carrying an effector gene based on RNAi and the red fluorescence protein (*DsRed*) reporter gene as the phenotypic marker gene was assessed and compared with that of their wild-type siblings. The authors found a comparative reduction in oviposition, fecundity, and adult lifespan, despite the longer lifespan for larvae, in the transgenic individuals than in their wild-type counterparts. In spite of the fitness cost, further study has shown that the fitness of these transgenic mosquitoes can be improved by treatment with the antibiotics co-trimoxazole, amoxicillin, and doxycycline (21). The important aspect of these studies is that they maintained the transgenic lines as hemizygotes/heterozygotes and that the fitness cost was directly due to transgenesis (i.e., expression of transgenes and insertional mutagenesis) rather than to the genetic background of the transgenic mosquitoes.

### Cage invasion experiments

The third approach is based on cage invasion studies, in which individuals carrying the transgenic allele are introduced into the wild population and the frequency of the gene is monitored over multiple generations, independent of the genetic background of the transgenic line. This mimics real release-like situations and, therefore, direct competition between transgenic and wild-type individuals can be observed in real-time. Moreover, this approach can be used to assess fitness in both hemizygotes (to determine the recessive–dominant effect) and homozygotes (to determine the dominant effect). In the light of these benefits, the fitness of four transgenic lines of *An. stephensi* expressin*g EGFP* or *DsRed* has been investigated in a cage invasion experiment in which an equal number of homozygous transgenic and non-transgenic mosquitoes were introduced and the fate of transgene in the population was studied (35). The authors found that the frequency of the transgenic allele in the cage population reduced sharply over time, and the allele became extinct after 4–16 generations. They later suggested that the loss of the transgenic allele could be explained by the reduced fitness cost of the inbred transgenic line, which originated from a single transformed mosquito crossed with one or a few wild-type mosquitoes. Any deleterious recessive mutation linked to the initial insertion can be fixed by creating a homozygous transgenic line (35). The aforementioned approaches, in combination, are now providing a better insight into the fitness of transgenic mosquitoes. For instance, determining the fitness parameters of transgenic mosquitoes, together with cage invasion studies, provides a better evaluation of the fitness of the mosquitoes. These combined fitness assessments are instrumental in defining fitness parameters required for population dynamic studies on the spread of transgenes in a targeted population by making cage invasion studies more effective (33). This approach has been used to study the fitness of three independent transgenic homozygous *An. stephensi* lines that produce salivary and midgut peptide 1 (SM1) effectors to inhibit the transmission of *Plasmodium berghei* (53). The authors, in a cage population experiment, assessed the life table parameters including egg hatchability, larvae to pupae viability, survival to adulthood, and mating success, along with transgene frequency changes (53). Life table analysis revealed a low fitness load in two single-insertion transgenic lines and no fitness load in a double-insertion transgenic line. The frequency of the transgene in all three transgenic lines decreased over time. The authors found that the reduction in transgene frequency is due not to instability but to the fact that male homozygotes compete less effectively for female counterparts, take a longer time to develop to adulthood, and have a lower fecundity than wild-type mosquitoes.

Despite the fact that numerous studies have examined the fitness of transgenic mosquitoes, only a few have so far shown transgenic lines to be associated with fitness advantages, or at least minimum fitness load, such that the transgene is stable and persists in the population over multiple generations. The combined efforts of a cage invasion experiment and comparison of the fitness parameters of heterozygous transgenic *An. stephensi* carrying two different effectors [a tetramer of the SM1 dodecapeptide and the phospholipase A_2_ gene (*PLA_2_*) from the honeybee] identified a significantly reduced fitness load in *PLA_2_* transgenics, but not in SM1 transgenics (52). Moreover, the authors found comparable fitness performance in two independent lines of SM1, suggesting that the position of the insertion has no effect on fitness. The fitness advantage of the SM1 mosquitoes over non-transgenic mosquitoes was due to the higher fecundity and lower mortality. The transgenic mosquitoes in this experiment were always maintained as heterozygotes to prevent the fixation of deleterious recessive alleles. However, the fact that the frequency of the transgenic allele increased in the population suggests that fitness is likely to be determined by overdominance (i.e., heterozygote superiority), in other words, that only a single wild-type allele would be enough to cover the deleterious recessive allele linked to the transgene on heterozygotes.

## Improvements to the fitness assessments

### Site-specific integration systems

Many experiments are currently underway to assess the fitness of transgenic mosquitoes in which various improvements and modifications to minimize the fitness load have been introduced. Of these, the site-specific transgene integration system φC31 is an efficient approach for the precise targeting of transgenes to predefined genomic sites. Integration systems comprise two phases. In phase 1, a transposable element is used to introduce one of the recombinase target sequences as a docking site, and in phase 2 a recombination enzyme is used to insert a transgene into the docking site. The power of this approach lies in the possibility of effectively generating and comparing multiple loaded transgenic lines from a single well-characterized docking site. Therefore, it enables effective control of the potential fitness load caused by random insertional mutagenesis and positional effects by enabling different effector genes and their regulatory sequences to be positioned precisely in the same location in the mosquito genome (33, 36). In a study by Paton *et al.* (33), the fitness of two transgenic lines of *An. gambiae* was assessed using a two-phase targeted site-specific transgene transformation system: the phase 1 docking strain carries a gene construct (EE) consisting of the phenotypic marker *ECFP* gene, and the phase 2 strain carries a gene construct (EVida3) consisting of the synthetic antimicrobial peptide (AMP) Vida3. The authors assessed reproductive success, mating, and hybrid vigor in the initial generation. In addition, a cage invasion experiment determined the frequency of transgenes in the EE and EVida3 genetic constructs independent of the strain’s original genetic backgrounds at first instar L1 larvae, pupae, and adult stages over 10 generations. Despite the fact that the overall genotypic fitness of phase 1 EE was comparable to that of the wild-type allele at all stages, the authors observed significantly lower allelic fitness in the transgenic strain relative to the wild-type allele during larval development. Moreover, a rapid reduction in the frequency of the EVida3 construct was observed within 10 generations, particularly during larval development, in both homozygous and hemizygous individuals. The significant reduction in larval development may be due to the unintended background expression of AMP and/or DsRed2 markers; however, this can be alleviated by carefully designing transgenic constructs and having them inserted into a site with a low background fitness cost.

### Gene drives

The use of gene drives has significantly increased interest in the genetic control of mosquitoes (56, 57). Conceptually, these drives exploit the innate DNA repair machinery of the organism to copy or home themselves into a target genomic site. This mechanism converts wild-type alleles into drive alleles in heterozygotes, thereby driving the super-Mendelian inheritance of the drive into succeeding generations regardless of the fitness cost to the organism. This inheritance mechanism can disseminate the drives and the desirable cargo genes, such as pathogen resistance, to fixation in a population in a very short time (58–60). The challenges in engineering homing drives with different endonucleases were resolved with the recent CRISPR/Cas9 revolution (61, 62). In addition to being an effective tool for genome editing, it can now be employed as a gene drive system. CRISPR-homing gene drives have recently been developed for mosquito control of two malaria vector species, *An. gambiae (* 63–65) and *An. stephensi (* 60 *)*, and a dengue control species, *Ae. aegypti (* 58 *).* These gene drive systems largely consist of two essential components: Cas9 endonucleases to facilitate gene drive integration into the genome and a guide RNA (gRNA) cassette, which encodes sequence-specific integration sites targeted by Cas9. A recent study by Li *et al. (* 58) evaluated the potential fitness cost associated with the gene drive components of *Ae. aegypti* in terms of fecundity, egg hatch rate, larval development time, male competitiveness, and adult survival. The authors found no significant differences in any of these fitness parameters between the transgenic and wild-type mosquito lines, with the exception of female fecundity. Reduced fecundity may be due to the expression of Cas9, which could be toxic, particularly when expressed at a high level. Furthermore, their mathematical models suggest that these gene drive systems could spread anti-pathogen effector genes into the wild in a safe, reliable, reversible manner and that they are suitable for field trials and effective for controlling diseases. These findings could expedite the development of transgenic mosquitoes that could safely control wild populations of mosquitoes to combat pathogen transmission. Another work by Kyrou *et al. (* 65), which employed the CRISPR/Cas9 gene drive *dsxF^CRISPRh^* targeting exon 5 of the doublesex (*dsx*) gene in *An. gambiae*, resulted in a completely sterile female. The authors conducted cage experiments in which heterozygous individuals bearing the *dsxF^CRISPRh^* allele were mixed with wild-type mosquito populations, and progeny were monitored at each generation to assess the spread of the drive, and to quantify its effects on reproductive output. The drive spread rapidly in caged mosquitoes, reaching 100% prevalence within 7–11 generations, while progressively reducing egg production to total population collapse. However, it is necessary to evaluate the gene drive in large, confined spaces more closely to mimic the natural environment (competition for food, presence of predators, and environmental stressors). The genetic makeup of the laboratory strain and the presence of the drive construct itself may cause heterozygous female mosquitoes harboring the drive allele to experience a further reduction in fitness. Despite this, gene drives with significant fitness costs in a population are more likely to become extinct despite a strongly biased gene drive inheritance.

## Future perspectives

Despite all these advancements, a major limitation of fitness assessments is that most have been conducted only in laboratory settings, and performance in the natural environment has not been adequately tested. Fitness estimates should be performed in the field because laboratory-based and field-based trials do not always produce comparable results. The environmental factors in the field may differ from those found in standard laboratory conditions. Consequently, transgenic mosquitoes in the field population will have to face different challenges following their release. Therefore, semi-field and field studies are of paramount importance in validating laboratory findings and gaining better insights into transgene fixation before releasing transgenic mosquitoes into the environment (66). For example, the field performance of the first-generation RIDL strain of *Ae. aegypti* OX513A males, developed by Oxitec (Abingdon, UK), has successfully been assessed in terms of mating competitiveness at Grand Cayman, British Overseas Territory, in the Caribbean, and Brazil. The study found that environmental and target strain differences had little impact on the mating success of the OX513A males, suggesting the ability of OX513A to reduce rates of disease transmission through population suppression (67–69). Subsequently, in field trials in Brazil, the second-generation RIDL strain of *Ae. aegypti* OX5034 also showed high levels of suppression (96%). Even more recently, open-air release has for the first time been permitted in Florida, USA, after decades of fighting for regulatory approval and public acceptance (70–72). However, continuous monitoring and testing of the fitness of *Ae. aegypti* OX513A is essential to ensure its success as a mosquito control program.

Despite the successful use of RIDL mosquitoes, fitness experiments among many other genetically modified mosquitoes made using other strategies remain limited to the laboratory and have not progressed beyond the field level owing to the fitness challenges. Therefore, new tools to minimize the fitness load are urgently required. However, there may always be some cost to fitness in practice when developing transgenic mosquitoes. Therefore, it is essential to assess fitness load and select the fittest transgenic line with the lowest possible fitness load. Self-limiting population reduction approaches always necessitate the release of a large number of transgenic mosquitoes to compensate for performance issues associated with mosquito fitness. Unlike the self-limiting population reduction strategies, self-sustaining replacement strategies are expected to continue the transgene in the targeted population indefinitely, addressing the need for transgenes to be coupled to an efficient gene drive system capable of stable transgene introgression into a natural population despite the fitness load (13).

## Conclusion

The genetic manipulation of mosquitoes as a control strategy during the past few years has shown its potential for sustainable and effective control of mosquito-borne diseases. These strategies generally rely on the mass release of transgenic mosquitoes into the wild, where the transgene is expected to persist in the environment for several generations or indefinitely. However, the transgene-associated fitness cost can greatly hinder the efficacy of these transgenic strategies. The major sources of fitness cost can mainly be categorized by the potential impact of the transgene expression, insertional mutagenesis, inbreeding depression related to laboratory adaptation, and the hitchhiking effect involved in developing homozygous mosquito lines. As the real estimate of transgene-associated fitness cost is of paramount importance to modeling and planning a transgenic mosquito release program, it is crucial to assess the fitness of transgenic mosquitoes before they are released, to make sure that they survive in the real environment. The fitness of transgenic mosquitoes can be assessed in several ways. First, the fitness parameters of homozygotes can be directly compared with that of their wild-type/unmodified parental strains; second, the fitness parameters of individuals hemizygous for transgene can be compared with those of their wild-type; third, and finally, cage invasion experiments can be used to monitor transgene frequency over time. All these approaches contribute to the development of different transgenic mosquitoes that will have minimum effect on fitness and to the assessment of the feasibility of using different transgenic strategies to make mosquito control programs more rapid and successful. Despite the enormous efforts to avoid fitness issues, only a handful of transgenic studies have shown fitness advantages, or at least fitness-neutral transformation that is stable in the environment for multiple generations, and there may always be some cost to the fitness of mosquitoes when producing transgenic lines. In this scenario, fitness issues can be overcome by the inundative release of a large number of mosquitoes. Alternatively, the transgenes can be linked to highly powerful gene drive mechanisms to overcome fitness cost, specifically in self-sustaining control strategies.

## Author contributions

PD is responsible for the writing of this article and the creation of the figure and table. PD, YS, and RD are responsible for the generation of the concepts and ideas provided. YS, RD, and BT are responsible for the editing of the manuscript. All authors contributed to the article and approved the submitted version.

## Funding

This work was supported by grant #6026-LK/8743-LK Accelerating Higher Education Expansion and Development (AHEAD) Operation.

## Conflict of interest

The authors declare that the research was conducted in the absence of any commercial or financial relationships that could be construed as a potential conflict of interest.

## Publisher’s note

All claims expressed in this article are solely those of the authors and do not necessarily represent those of their affiliated organizations, or those of the publisher, the editors and the reviewers. Any product that may be evaluated in this article, or claim that may be made by its manufacturer, is not guaranteed or endorsed by the publisher.
